# F41. SCHIZOPHRENIA-RELEVANT ALTERATIONS IN CEREBRAL METABOLISM, GLUTAMATE AND MONOAMINERGIC NEUROTRANSMITTER SYSTEM FUNCTION IN A MOUSE MODEL OF 16P11.2 DUPLICATION

**DOI:** 10.1093/schbul/sby017.572

**Published:** 2018-04-01

**Authors:** Greg Bristow, David Thomson, Judith Pratt, Brian Morris, Neil Dawson

**Affiliations:** 1 Lancaster University; 2 University of Strathclyde; 3 University of Strathclyde, Strathclyde Institute Pharmacy & Biomedical Science; 4 University of Glasgow

## Abstract

**Background:**

Duplication at 16p11.2, affecting approximately 30 genes, has consistently been associated with increased risk of schizophrenia (Psychiatric Genomics Consortium, 2017. Nat Genet 49: 27). We currently have little understanding of how this CNV impacts on brain and neurotransmitter system function. Here we use a mouse model of 16p11.2 duplication (7DUP mice, Horev et al. 2011. PNAS. 108: 17076) to determine the impact of this CNV on cerebral metabolism. In addition, we characterize in vivo glutamate and monoamine neurotransmitter system function by challenging these animals acutely with ketamine and d-amphetamine, respectively.

**Methods:**

7DUP mice and littermate controls were treated with ketamine (25mg/kg), d-amphetamine (5mg/kg), or saline (2ml/kg). n=11 (6 male, 5 female) for each genotype per treatment group. Cerebral metabolism was determined by 14C-2-deoxyglucose functional brain imaging (Dawson et al., 2015.Transl Psychiatry. 5:e569). Data were analysed using repeated measures ANOVA with post-hoc Tukey’s HSD.

**Results:**

7DUP mice show significant constitutive hypometabolism in the thalamic reticular nucleus, mesolimbic system, and in neuromodulatory brain regions. Hypometabolism was also seen in the striatum of female, but not in male, 7DUP mice. 7DUP mice were also found to show hypermetabolism in the hippocampus, amygdala, and cerebral cortex. The impact of ketamine on cerebral metabolism is attenuated in 7DUP mice with sex specific effects, being evident in the mesolimbic and neuromodulatory system of males, whereas the attenuation is present in the hippocampus and striatum in female mice. By contrast, 7DUP mice showed an exaggerated response to d-amphetamine. Again, these effects were influenced by sex, with the exaggerated response being significantly more widespread in males than in females.

**Discussion:**

7DUP mice show altered constitutive cerebral metabolism in brain regions implicated in schizophrenia, including hippocampal and temporal cortex hyperactivity. In addition, 7DUP mice demonstrate a reduced response to ketamine, supporting NMDA receptor hypofunction as a result of 16p11.2 duplication. This effect is consistent with the glutamate hypofunction hypothesis of schizophrenia. By contrast, 7DUP mice show an exaggerated response to d-amphetamine, supporting monoamine neurotransmitter system dysfunction as a consequence of 16p11.2 duplication. Intriguingly, each of these effects differs in male and female mice, suggesting that the phenotypic impact of 16p11.2 duplication is influenced by sex. These data provide new insight into the mechanisms through with 16p11.2 duplication increases the risk of developing schizophrenia.

